# Supplementary material for: Living Material with Temperature‐Dependent Light Absorption
Source: Adv Sci (Weinh). 2023 Sep 15;10(30):2301730. doi: 10.1002/advs.202301730 (PMC10602556; doi:10.1002/advs.202301730)
Supplement: Supplementary file 1 — Supporting Information [file ADVS-10-2301730-s001.pdf]

ADVANCED SCIENCE
Open Access

Supporting Information

for *Adv. Sci.*, DOI 10.1002/advs.202301730

Living Material with Temperature-Dependent Light Absorption

Lealia L. Xiong, Michael A. Garrett, Julia A. Kornfield and Mikhail G. Shapiro**

Supporting information

Living material with temperature-dependent light absorption

Lealia L. Xiong[1], Michael A. Garrett[2], Julia A. Kornfield[2,*], Mikhail G. Shapiro[2,3,*]

[1] Division of Engineering and Applied Sciences
[2] Division of Chemistry and Chemical Engineering
[3] Howard Hughes Medical Institute
California Institute of Technology
Pasadena, CA USA 91125

* Correspondence should be addressed to MGS and JAK: mikhail@caltech.edu, jak@cheme.caltech.edu

Table of contents

Supplementary Tables S1-S2
Supplementary Figures S1-S12

SUPPLEMENTARY TABLES S1-S2

Supplementary Table S1. Genetic constructs used in this study.

**Plasmid**	**Purpose**	**Transcriptional Regulator(s)**	**Output Gene Product(s)**
pTSwitch-LacZα	temperature switch	TlpA36, CI	LacZα, mWasabi
pTlpA36-wasabi	unpigmented control	TlpA36	mWasabi
pTrcLacZα	pigmented control	LacIq	LacZα

Supplementary Table S2. Sequences of DNA parts used in this study.

Type	Name	Sequence
promoter	PTlpA	TTTAATTTGTTTGTTAGTTAGTTTATTTGTTGGTTTGTTTGTGTTATA<br>ATAT
promoter	PR	GTGCGTGTTGACTATTTTACCTCTGGCGGTGATAATGGTTGCATGTACTAAG<br>GAGGTTG
promoter	PL	AACCATCTGCGGTGATAAATTATCTCTGGCGGTGTTGACATAAATACCACTG<br>GCGGTGATACTGAGCACATCAGCAGG
promoter	PTrc	TTGACAATTAATCATCCGGCTCGTATAATGTGTGGAATTGTGAGCGGATAAC<br>AATT
degradation tag	AAV ssrA tag	GCTGCTAACGACGAAAACTACGCTGACGCTTCT
terminator	T7	CTAGCATAACCCCTTGGGGCCTCTAAACGGGTCTTGAGGGGTTTTTTG
terminator	Part:BBa_B1002	CGCAAAAAACCCCGCTTCGGCGGGGTTTTTTCGC
RBS	RBSF	CACCATACACTG
gene	LacZa	CATGATTACGGATTCACTGGCCGTCGTTTTACAACGTCGTGACTGGGAAAAC<br>CCTGGCGTTACCCAACTTAATCGCCTTGCAGCACATCCCCCTTTCGCCAGCT<br>GGCGTAATAGCGAAGAGGCCCGCACCGATCGCCCTTCCCAACAGTTGCGCA<br>GCCTGAATGGCGAATGGCGCTTTGCCTGGTTTCCGGCACCAGAAGCGGTGC<br>CGGAAAGCTGGCTGGAG
gene	mWasabi	ATGGTGAGCAAGGGCGAGGAGACCACAATGGGCGTAATCAAGCCCGACATG<br>AAGATCAAGCTGAAGATGGAGGGCAACGTGAATGGCCACGCCTTCGTGATCG<br>AGGGCGAGGGCGAGGGCAAGCCCTACGACGGCACCAACACCATCAACCTGG<br>AGGTGAAGGAGGGAGCCCCCCTGCCCTTCTCCTACGACATTCTGACCACCGC<br>GTTCAGTTACGGCAACAGGGCCTTCACCAAGTACCCCGACGACATCCCCAAC<br>TACTTCAAGCAGTCCTTCCCCGAGGGCTACTCTTGGGAGCGCACCATGACCT<br>TCGAGGACAAGGGCATCGTGAAGGTGAAGTCCGACATCTCCATGGAGGAGG<br>ACTCCTTCATCTACGAGATACACCTCAAGGGCGAGAACTTCCCCCCCAACGG<br>CCCCGTGATGCAGAAGGAGACCACCGGCTGGGACGCCTCCACCGAGAGGAT<br>GTACGTGCGCGACGGCGTGCTGAAGGGCGACGTCAAGATGAAGCTGCTGCT<br>GGAGGGCGGCGGCCACCACCGCGTTGACTTCAAGACCATCTACAGGGCCAA<br>GAAGGCGGTGAAGCTGCCCGACTATCACTTTGTGGACCACCGCATCGAGATC<br>CTGAACCACGACAAGGACTACAACAAGGTGACCGTTTACGAGATCGCCGTGG<br>CCCGCAACTCCACCGACGGCATGGACGAGCTGTACAAGGGC
gene	TlpA36	ATGCGTCCGGCGACATACGAACCAGAACAGATTATTGAAGCAGGGCTGGCCC<br>TGCAGGCTGAAGGACGGAATATCACCGGGTTCGCACTACGTAACCAGGTGG<br>GTGGCGGCAATCCGACACGTCTCCGCCAGATATGGGACGAATACCAGGCTT<br>CACAGAGCACGGTCGTCACTGAACTCGTTGCCGAGCTGCCAGTGGAAGTGG<br>CTGAAGAAGTGAAGGCCGTCTCCGCCGCGCTGTCCGAACGCATCACCCAGC<br>TGGCGACAGAACTGAATGACAAGGCGGTCCGGGCTGCAGAACGCCGGGTTG<br>CGGAAGTCACGCGTGCTGCCGGTGAACAGACCGCACAGGCAGAGCGGGAGC<br>TGGCCGACGCCGCGCAGACAGTCGACGACCTGGAAGAAAAACTGGTTGAACT<br>GCAGGACAGATATGACAGTTTGACGCTGGCGCTGGAGTCAGAACGTTCACT<br>GCGTCAGCAGCATGATGTGGAGATGGCCCAGCTGAAAGAGCGTCTTGCGGC<br>CGCTGAAGAGAATACCCGTCAGCGAGAGGAACGGTATCAGGAGCAGAGGAC<br>AGTGCTGCAGGATGCGCTTAATGCGGAGCAGGCACAGCACATAAACACGCG<br>GGAAGACCAGCAGAAACGACTGGAGCAAATTTCTGCCGAAGCTAATGCGCGT<br>ACAGAAGAACTGAAGTCTGAACGCGATAAAGTCAATACTCTCCTTACCCGCC<br>TTGAATCGCAGGAAAATGCGCTGGCCTCAGAACGTCAGCAGCATCTGGCCAC<br>CCGCGAAACGCTGCAGCAACGCCTCGAGCAGGCCATCGCTGACACGCAGGC<br>GCGCGCCGGTGAGATTGCACTTGAACGTGACAGAGTCAGCAGCCTCACCGC<br>AAGGCTGGAATCGCAGGAAAAGGCCTCCTCGGAGCAACTGGTGCGTATGGG<br>CAGTGAAATAGCCAGTCTGACAGAGCGTTGCACACAGCTGGAAAACCAGCGT<br>GATGATGCCCGTCTGGAGACGATGGGGGAGAAAGAAACGGTCGCGGCACTG<br>CGTGGTGAGGCTGAAGCCCTGAAGCGTCAGAACCAGTCACTGATGGCGGCG<br>CTTTCAGGCAATAAACAGACCGGTGGCCAGAATGCGT

gene	CI	ATGAGCACAAAAAAGAAACCATTAACACAAGAGCAGCTTGAGGACGCACGTC<br>GCCTTAAAGCAATTTATGAAAAAAAGAAAAATGAACTTGGCTTATCCCAGGAA<br>TCTGTCGCAGACAAGATGGGGATGGGGCAGTCAGGCGTTGGTGCTTTATTT<br>AATGGCATCAATGCATTAAATGCTTATAACGCCGCATTGCTTGCAAAAATTCT<br>CAAAGTTAGCGTTGAAGAATTTAGCCCTTCAATCGCCAGAGAAATCTACGAG<br>ATGTATGAAGCGGTTAGTATGCAGCCGTCACTTAGAAGTGAGTATGAGTACC<br>CTGTTTTTTCTCATGTTCAGGCAGGGATGTTCTCACCTGAGCTTAGAACCTT<br>TACCAAAGGTGATGCGGAGAGATGGGTAAGCACAACCAAAAAAGCCAGTGAT<br>TCTGCATTCTGGCTTGAGGTTGAAGGTAATTCCATGACCGCACCAACAGGCT<br>CCAAGCCAAGCTTTCCTGACGGAATGTTAATTCTCGTTGACCCTGAGCAGGC<br>TGTTGAGCCAGGTGATTTCTGCATAGCCAGACTTGGGGGTGATGAGTTTAC<br>CTTCAAGAAACTGATCAGGGATAGCGGTCAGGTGTTTTTACAACCACTAAAC<br>CCACAGTACCCAATGATCCCATGCAATGAGAGTTGTTCCGTTGTGGGGAAAG<br>TTATCGCTAGTCAGTGGCCTGAAGAGACGTTTGGCTGA

SUPPLEMENTARY FIGURES S1-S11

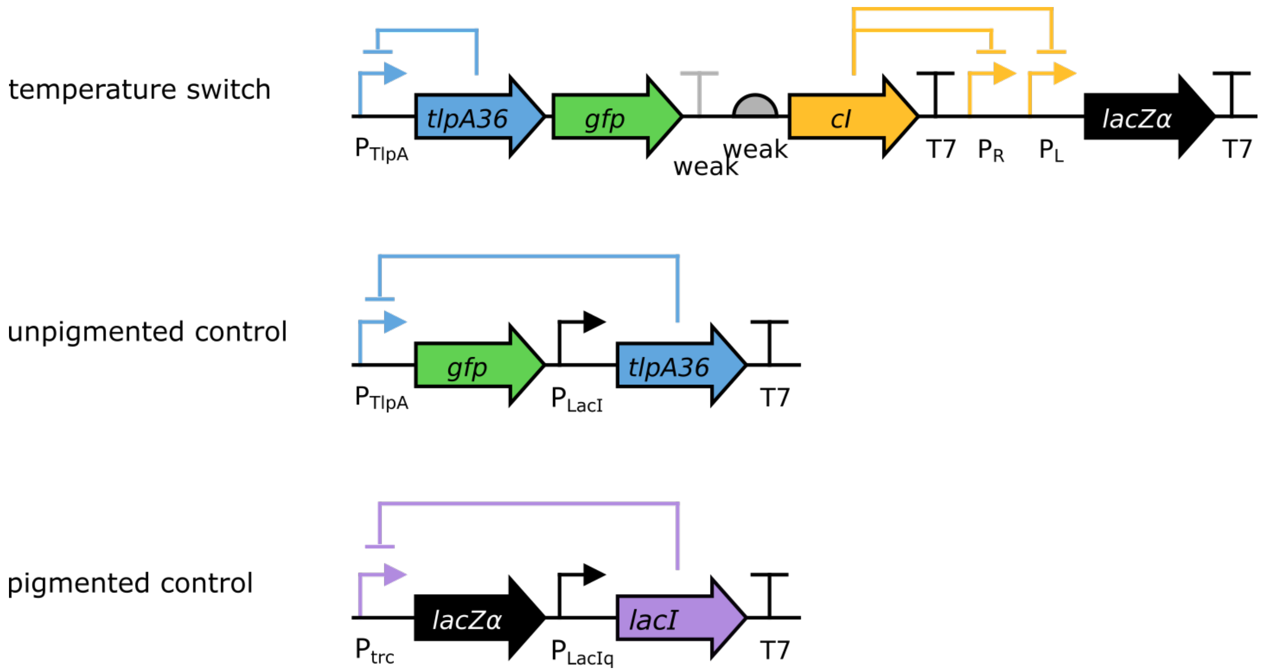

Supplementary figure S1. Circuit diagrams of temperature switch contruct for low-temperature pigmentation, unpigmented control, and pigmented control. The unpigmented control construct encodes mWasabi green fluorescent protein (GFP) under the control of TlpA36. Below 36°C, TlpA36 represses the expression of GFP by binding to the P_{TlpA} promoter. Above 36°C, TlpA36 loses repressor function, so GFP is expressed from the P_{TlpA} promoter. The pigmented control construct encodes LacZα peptide under the control of the *lac* repressor (LacI). LacI represses the expression of LacZα by binding to the P_{trc} promoter. Isopropyl β-D-1-thiogalactopyranoside (IPTG) binds to LacI and causes an allosteric change in its shape, causing LacI to lose its ability to bind to the P_{trc} promoter. Thus, IPTG induces the expression of LacZα from this contruct.

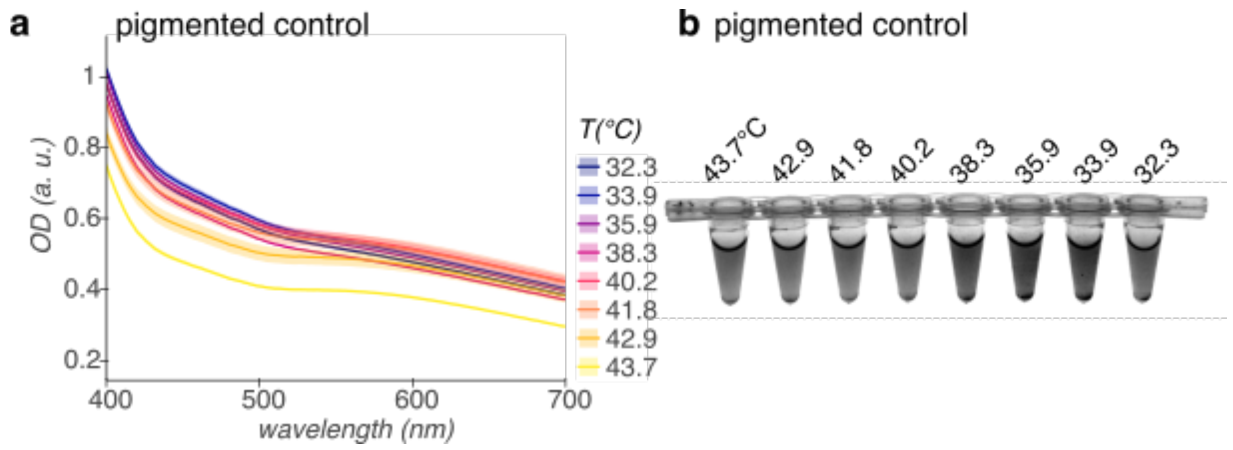

Supplementary figure S2. Visible light optical density (OD) spectra **(a)** and representative white light transillumination image **(b)** of cultures of *E. coli* containing a pigmented control construct encoding IPTG-inducible LacZα after 24 h growth in pigment-induction media at temperatures ranging from 43.7°C to 32.3°C. n = 2 biological replicates; shading represents +/- standard error of the mean.

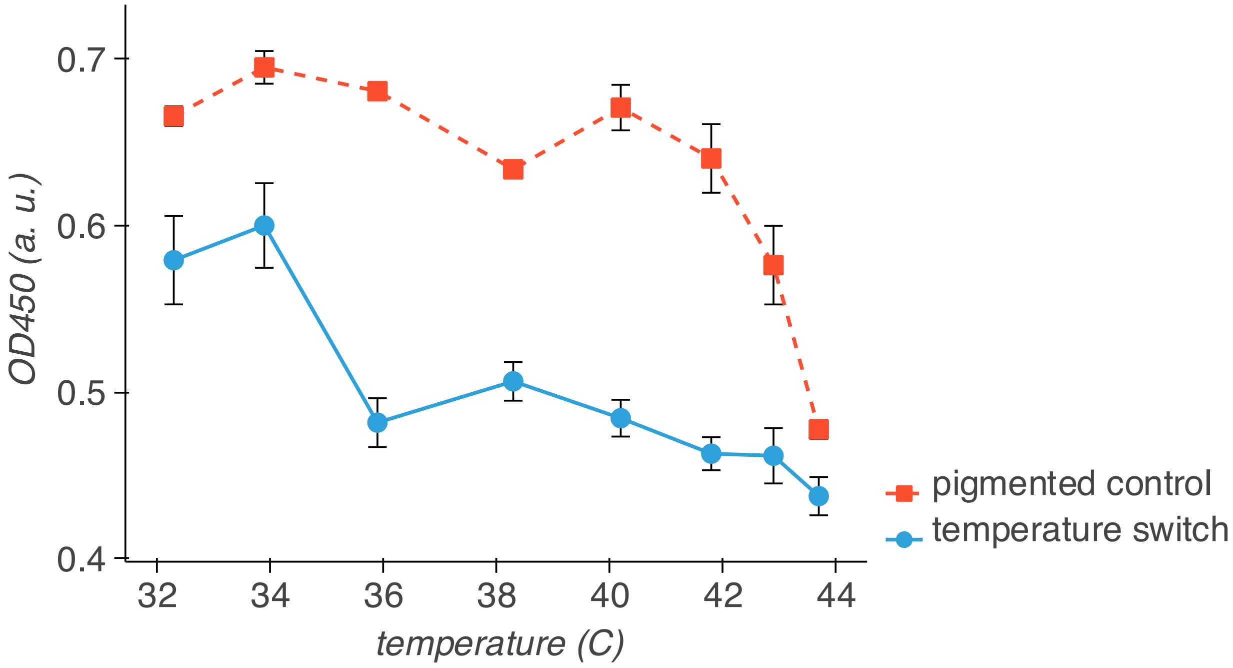

Supplementary figure S3. Optical density at 450 nm of cultures of *E. coli* containing the temperature switch construct (n = 4) or the pigmented control construct encoding IPTG-inducible LacZα (n = 2) after 24 h growth in pigment-induction media at temperatures ranging from 43.7°C to 32.3°C. 450 nm is within the visible light spectrum, but avoids the maximum excitation wavelength of mWasabi, 493 nm.

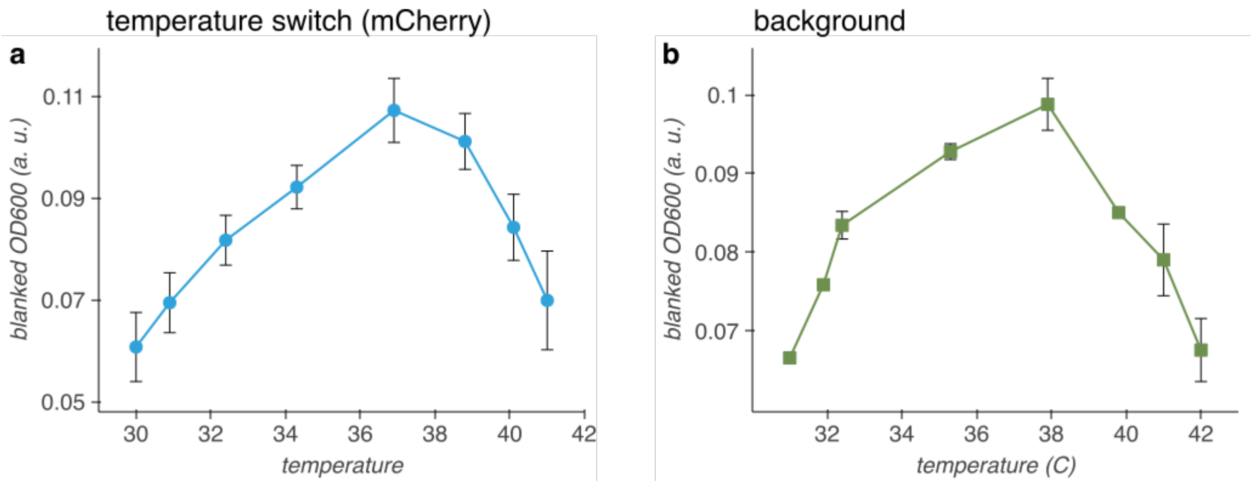

Supplementary figure S4. Cell density of *E. coli* grown for 12 hours at temperatures ranging from 31°C to 42°C, measured by optical density at 600 nm (OD600). **(a)** OD600 of cultures of DH10B *E. coli* containing a variant of the temperature switch circuit wherein *mCherry* replaces *lacZα*. *n* = 4 biological replicates; error bars represent +/- standard error of the mean. **(b)** OD600 of cultures of DH10B *E. coli* containing a construct encoding a nonfluorescent mutant of mWasabi (S71T, G73A) under the control of TlpA. At these temperatures, TlpA is always functional, repressing the expression of the nonfluorescent mWasabi. *n* = 2 biological replicates; error bars represent +/- standard error of the mean.

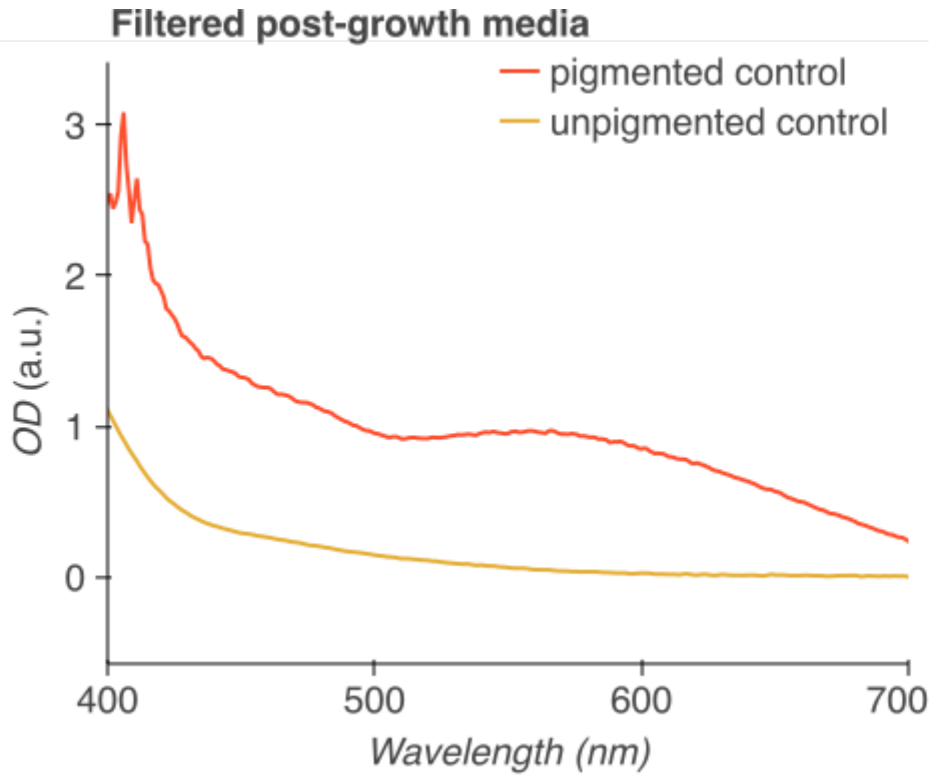

Supplementary figure S5. Visible light optical density (OD) spectra of media from *E. coli* cultures grown for 23 h at 30°C, 250 rpm with 300 μg/mL S-gal, 1 h after removing cells by passing through a 0.22 μm filter and adding 500 μg/mL ferric ammonium citrate. Black pigment forms visibly in media from *E. coli* containing a pigmented control circuit, resulting in increased optical density from 450 nm to 700 nm compared with media from *E. coli* containing an unpigmented control circuit. Spectra measured using a NanoDrop 2000/2000c Spectrophotometer (ThermoFisher) in cuvette mode. Constructs differ from main text slightly: Unpigmented control encodes nonfluorescent mWasabi mutant under the control of TlpA (TlpA represses product at 30°C). Pigmented control encodes IPTG-inducible full-length LacZ. Temperature switch construct lacks AAV ssrA tag on LacZα.

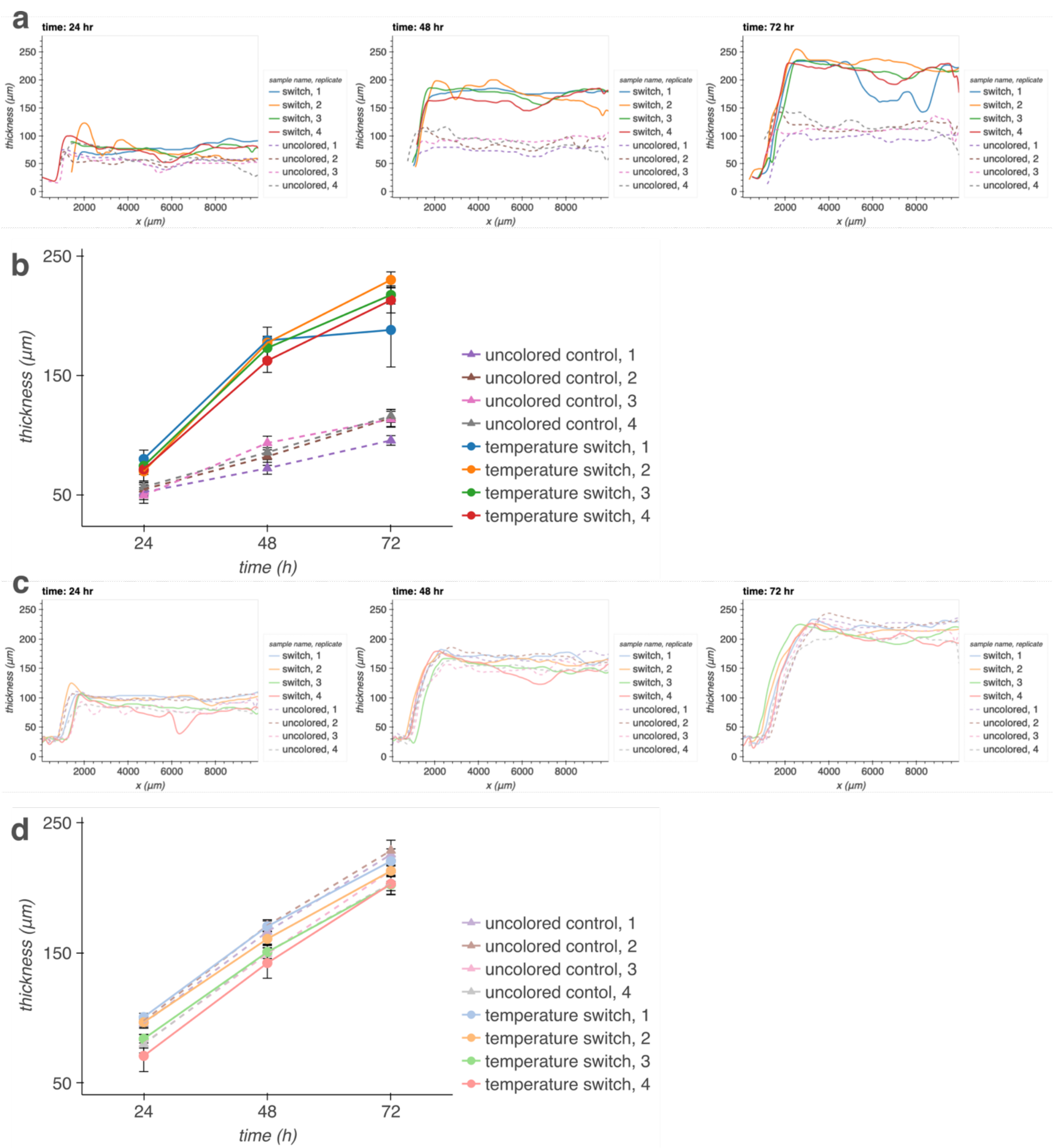

Supplementary figure S6. (**a, c**) Thicknesses over time measured across full OCT cross-section for each patch of *E. coli* containing either the temperature switch construct or an unpigmented control construct encoding heat-inducible GFP, grown at 32°C with (**a**) or without (**c**) illumination. (**b, d**) Mean thickness and standard deviation between x = 3.5 mm and x = 9.0 mm (avoiding the edges of the patch) of each patch, grown with (**b**) or without (**d**) illumination over time.

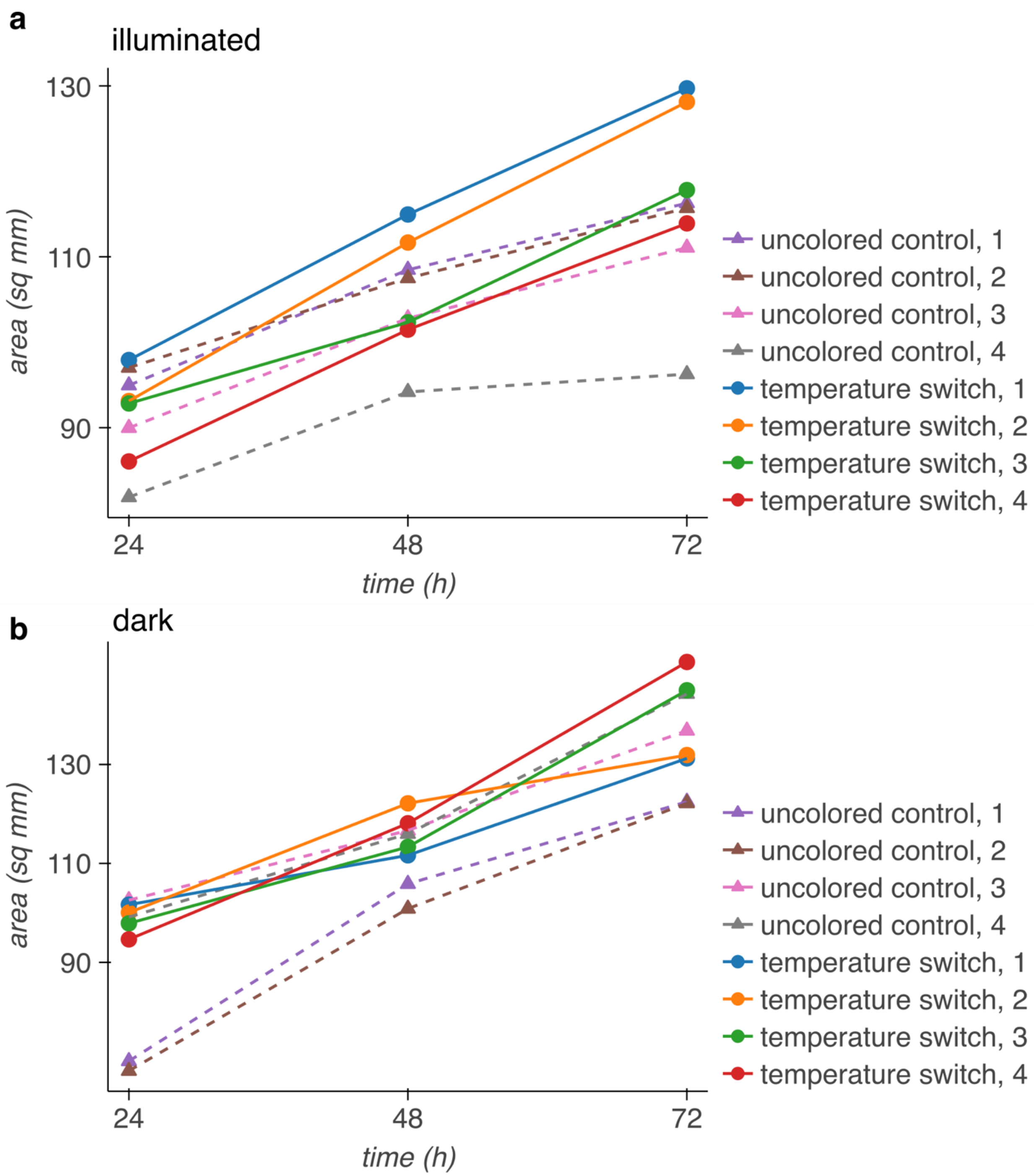

Supplementary figure S7. Area of patches of *E. coli* containing either the temperature switch construct or an unpigmented control construct encoding heat-inducible GFP, grown at 32°C with (**a**) or without (**b**) illumination over time.

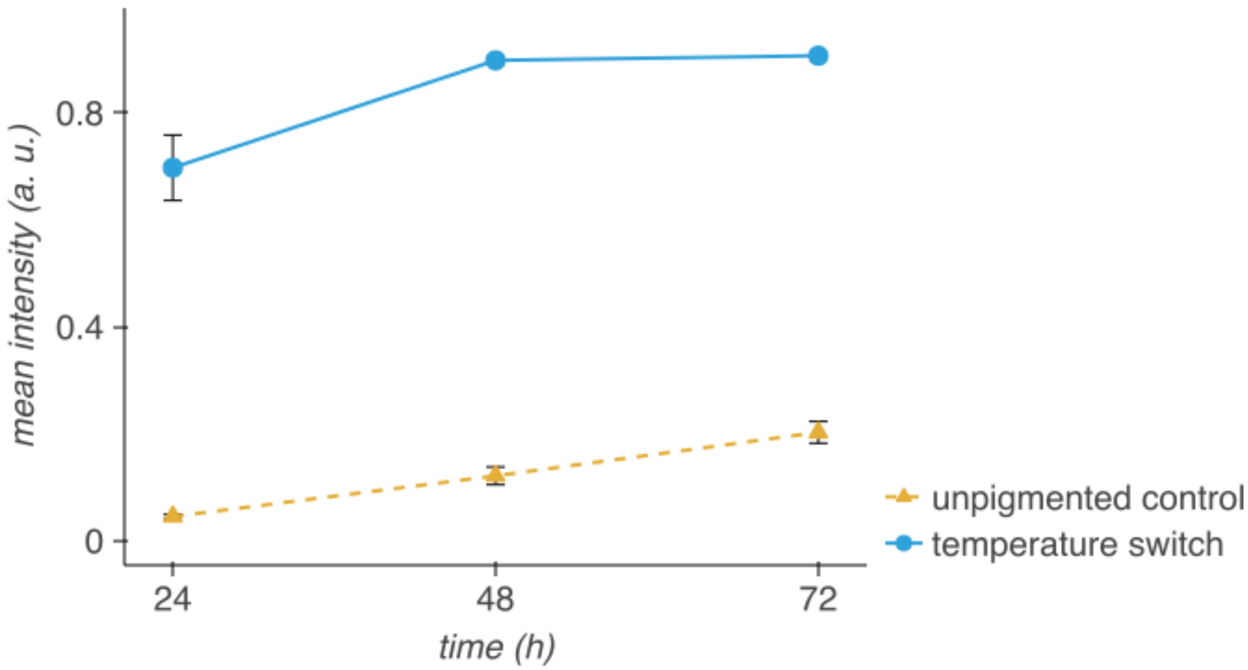

Supplementary figure S8. Mean pixel intensity of patches of *E. coli* containing either the temperature switch construct or an unpigmented control construct encoding heat-inducible GFP, grown at 32°C under illumination, over time. Data was quantified from a series of transillumination. Images were normalized so that the polycarbonate membranes have a mean intensity of 0 and opaque black plastic has a mean intensity of 1. *n* = 4 biological replicates; error bars represent +/- standard error of the mean.

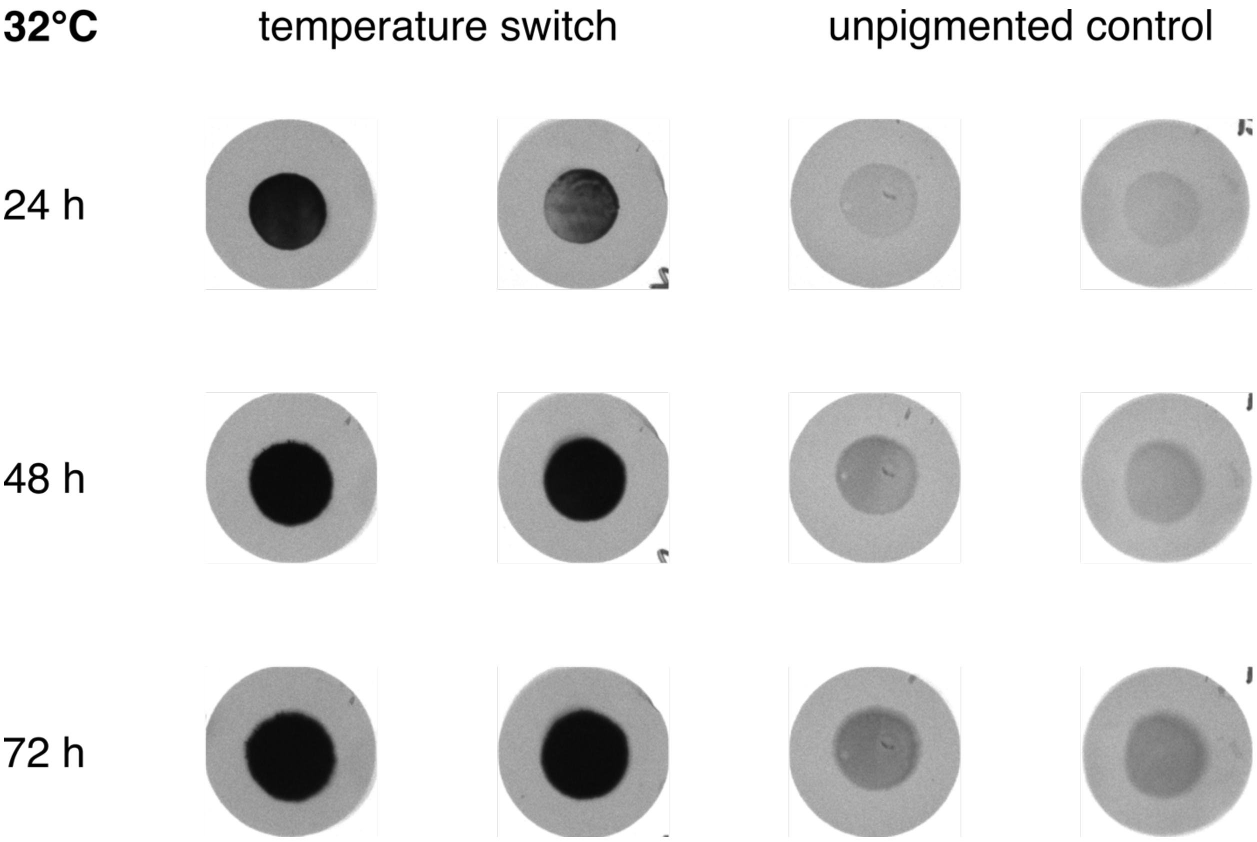

Supplementary figure S9. Representative transillumination white light images of patches of *E. coli* containing the temperature switch construct or the unpigmented control construct on polycarbonate membranes after 24 h, 48 h, and 72 h of growth in the illuminated growth chamber with pigment-induction media at 32°C.

r

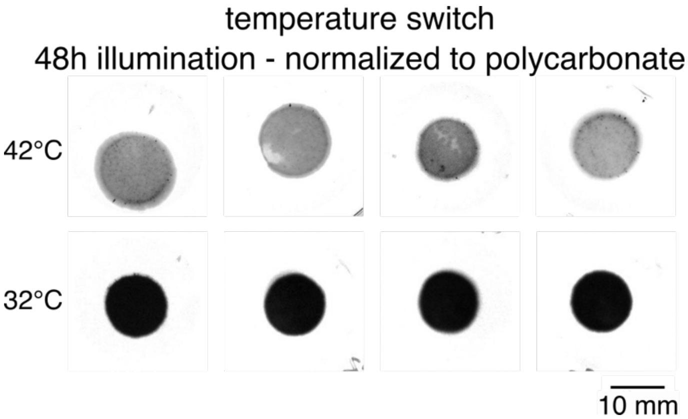

Supplementary figure S10. Transillumination white light images of patches of *E. coli* containing the temperature switch construct on polycarbonate membranes after 48 h growth in the illuminated growth chamber with pigment-induction media at 42°C and 32°C, normalized so that the polycarbonate membranes have a mean intensity of 0 and opaque black plastic has a mean intensity of 1.

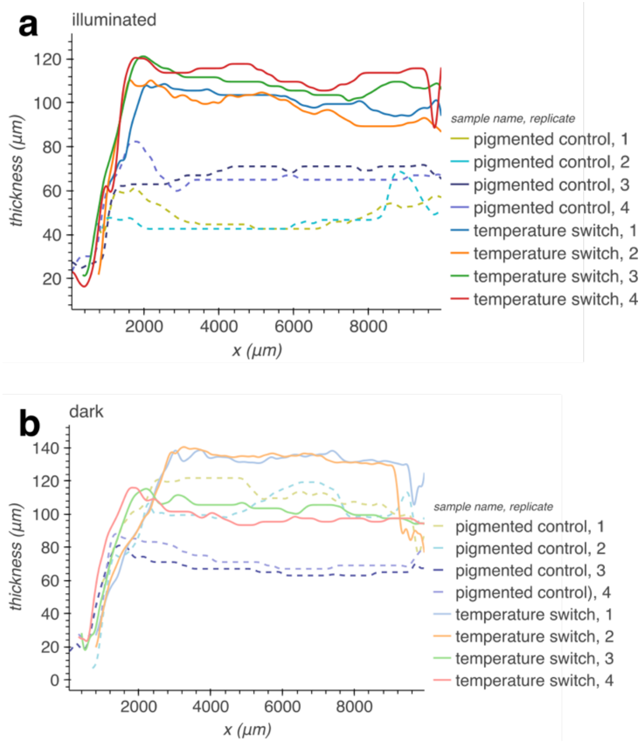

Supplementary figure S11. Thicknesses measured across full OCT cross-section for each patch of *E. coli* containing either the temperature switch construct or a pigmented control construct encoding IPTG-inducible LacZα, grown at 42°C for 48 h with **(a)** or without **(b)** illumination. Replicates 1 and 2 for both the pigmented control and the temperature switch construct were coated on Whatman Nuclepore polycarbonate membranes. Replicates 3 and 4 were coated on Sartorius polycarbonate membranes.

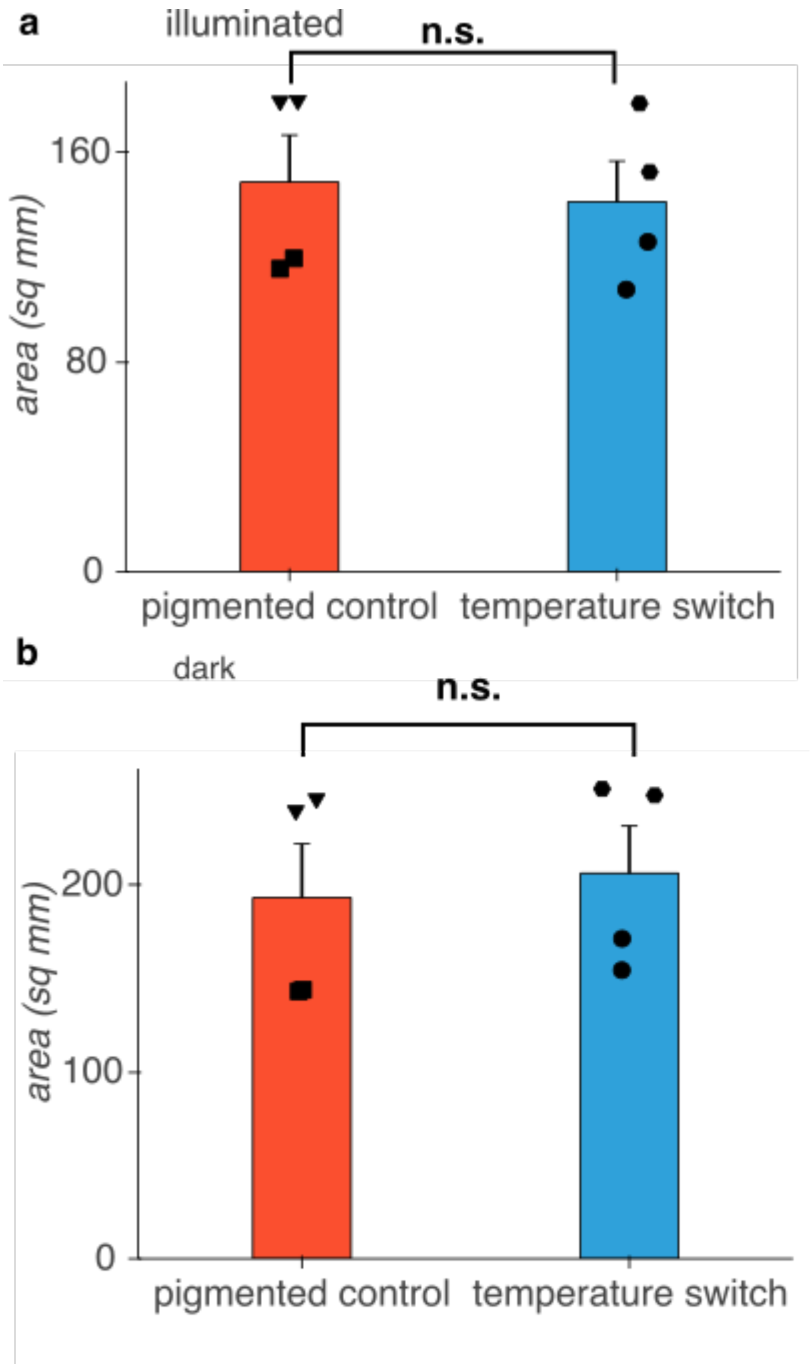

Supplementary figure S12. Area of patches of *E. coli* containing either the temperature switch construct or a pigmented control construct encoding IPTG-inducible LacZα, grown at 42°C with (**a**) or without (**b**) illumination for 48 h. Inverted triangle and hexagon markers indicate patches coated onto Whatman Nucleopore polycarbonate membranes; square and circle markers indicate patches coated onto Sartorius polycarbonate membranes.

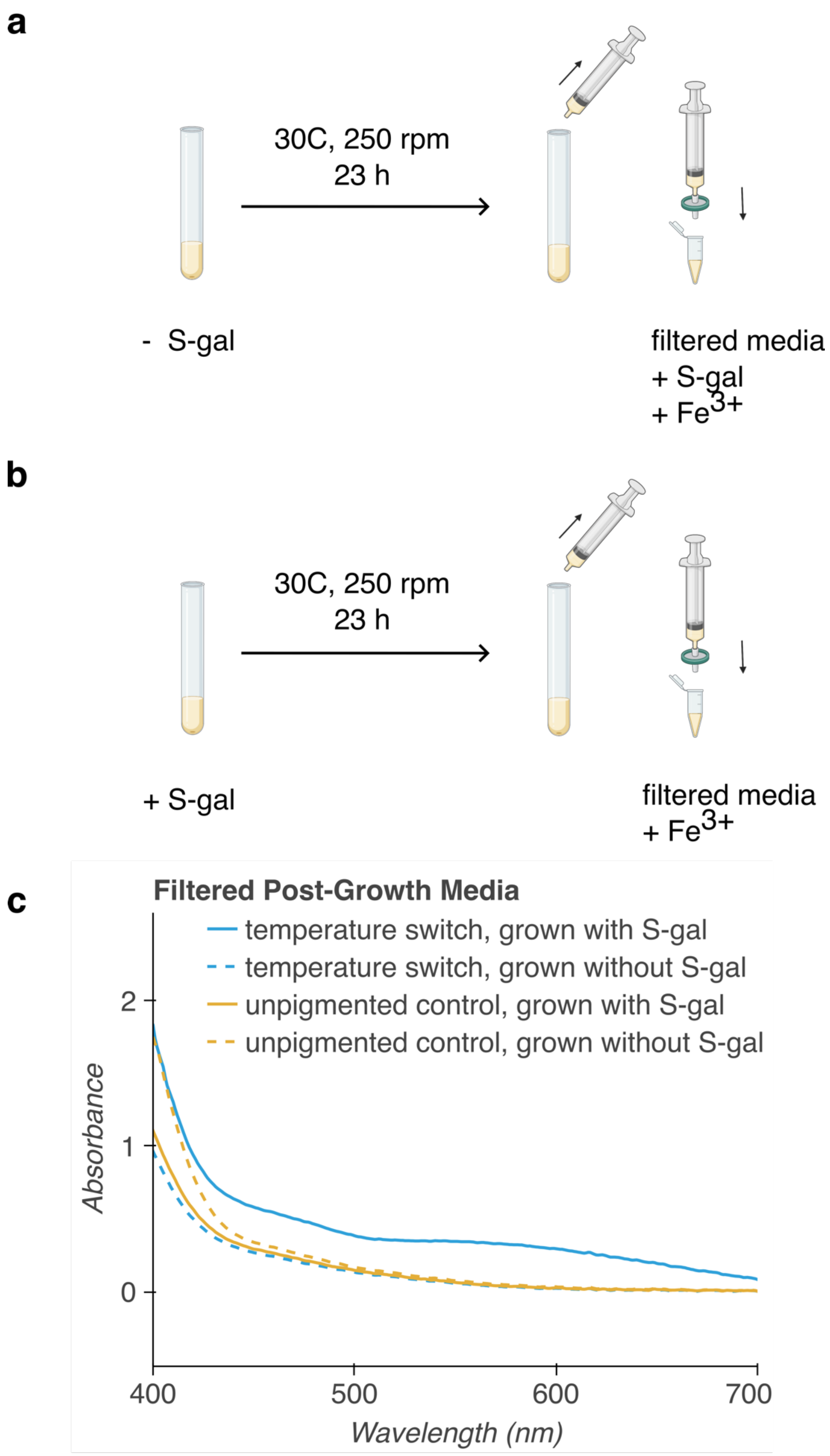
a
30C, 250 rpm
23 h
- S-gal
filtered media
+ S-gal
+ Fe3+
b
30C, 250 rpm
23 h
+ S-gal
filtered media
+ Fe3+
c
Filtered Post-Growth Media
temperature switch, grown with S-gal
temperature switch, grown without S-gal
unpigmented control, grown with S-gal
unpigmented control, grown without S-gal
Absorbance
2
1
0
400
500
600
700
Wavelength (nm)

Supplementary figure S13. Enzymatic cleavage of S-gal occurs intracellularly before extracellular coordination with ferric iron to form pigment. (**a**) DH10B *E. coli* were grown in LB media supplemented with 100 μg/mL ampicillin at 30°C, 250 rpm for 23 h. The cultures were passed through a 0.22 μm filter to remove the cells; then, 300 μg/mL S-gal and 500 μg/mL ferric ammonium citrate were added to the filtered media. (**b**) DH10B *E. coli* were grown in LB media supplemented with 100 μg/mL ampicillin and 300 μg/mL S-gal at 30°C, 250 rpm for 23 h. The cultures were passed through a 0.22 μm filter to remove the cells; then, 500 μg/mL ferric ammonium citrate were added to the filtered media. (**c**) Visible light optical density spectra of cultures of *E. coli* 1 h after filtering and adding either S-gal or S-gal and ferric ammonium citrate. The temperature switch sample grown with S-gal exhibits increased optical density from 450 nm to 700 nm compared with the unpigmented control sample grown with and without S-gal and the temperature switch sample grown without S-gal. This suggests that β-galactosidase cleaves S-gal intracellularly and at least some of the cyclohexenoesculetin product is exported from the cell, where it coordinates with ferric iron to form the light-absorptive pigment. Spectra measured using a NanoDrop 2000/2000c Spectrophotometer (ThermoFisher) in cuvette mode. Constructs differ from main text slightly: Unpigmented control encodes nonfluorescent mWasabi mutant under the control of TlpA (TlpA represses product at 30°C). Pigmented control encodes IPTG-inducible full-length LacZ. Temperature switch construct lacks AAV ssrA tag on LacZα.
